# Pattern Formation on Networks: from Localised Activity to Turing Patterns

**DOI:** 10.1038/srep27397

**Published:** 2016-06-07

**Authors:** Nick McCullen, Thomas Wagenknecht

**Affiliations:** 1 Centre for Networks and Collective Behaviour, Architecture & Civil Engineering, University of Bath, UK; 2 Department of Applied Mathematics, University of Leeds, UK

## Abstract

Networks of interactions between competing species are used to model many complex systems, such as in genetics, evolutionary biology or sociology and knowledge of the patterns of activity they can exhibit is important for understanding their behaviour. The emergence of patterns on complex networks with reaction-diffusion dynamics is studied here, where node dynamics interact via diffusion via the network edges. Through the application of a generalisation of dynamical systems analysis this work reveals a fundamental connection between small-scale modes of activity on networks and localised pattern formation seen throughout science, such as solitons, breathers and localised buckling. The connection between solutions with a single and small numbers of activated nodes and the fully developed system-scale patterns are investigated computationally using numerical continuation methods. These techniques are also used to help reveal a much larger portion of of the full number of solutions that exist in the system at different parameter values. The importance of network structure is also highlighted, with a key role being played by nodes with a certain so-called optimal degree, on which the interaction between the reaction kinetics and the network structure organise the behaviour of the system.

## Patterns on networks

Patterns are found throughout nature and much of science is dedicated to identifying and understanding the origin and growth of such patterns. Alan Turing first developed a mathematical theory of pattern formation for spatial media in an attempt to explain cellular differentiation and morphogenesis^1^. His analysis looked at a symmetry breaking bifurcation in reaction-diffusion systems of partial differential equations (PDEs) in continuous media and subsequent research by Othmer and Scriven^2^ generalised this to discrete lattices in a framework that can be applied to other, potentially complex, network topologies.

Many natural and human systems can be represented as networks^3^,^4^, where individual elements are represented by nodes on a graph and interactions between them as edges. There is also growing interest in using models of reaction-diffusion systems organised on complex network topologies, particularly in systems with activator-inhibitor dynamics on the nodes, to explain interesting biological applications such as pattern development arising in networks of activating and suppressing genes involved in embryonic development^5^ or the evolution of complex structures (*autocatalytic sets*) in systems of competing proteins, such as could lead to the origin of life from a random starting condition^6^. However, the networks connecting such species, genes or individuals in a social system often have non-local connections^7^ and non-trivial topologies^8^,^9^, making the concept of a *pattern* less clear in such non-spatial domains. Recent numerical results have revealed a multiplicity of bulk activation states (referred to as *Turing modes*) in a predator-prey type reaction-diffusion model on a scale-free (*Barabási–Albert*) type network^5^.

The work here demonstrates how smaller-scale patterns of activity on random networks are closely related to localised patterns in continuous media such as solitons or localised buckling^10^,^11^,^12^. These are connected to increasingly larger patterns of activity and bulk-modes via a winding solution structure known as “snaking”^13^,^14^. Numerical methods used in dynamical systems on regular topologies are used to reveal much about the solution space and the multiplicity of coexisting states across the range of driving parameters. The importance of network structure in the dynamics is also demonstrated, with the important role played by the so-called *optimal degree*^15^ node playing a crucial role. The solution structure for these systems have a highly complex *“turmoil”* of coexisting states, necessitating a statistical approach to understanding the behaviour of the system.

## Pattern formation and reaction-diffusion systems

Alan Turing laid down the basis for pattern formation on a spatial domain, based the loss of stability of an unpatterned equilibrium to another non-trivial (patterned) state^1^, in an attempt to explain morphogenesis in embryonic development. Such situations can be set up as a system of competing chemical species in a reaction-diffusion system of PDEs:


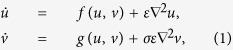


where *u* and *v* are activator and inhibitor chemical species, *f* and *g* are functions for the internal *reaction* component at any location and ∇^2^ is the Laplacian diffusion operator. Such formulations have been widely used to describe pattern formation in a wide variety of systems on a spatial domain. In numerical investigations of such systems space is discretised, with the reactions taking place on nodes on a regular mesh and diffusion occurring to neighbouring nodes via local network edge connections on the lattice. In these cases the diffusion operator is replaced with the discrete Laplacian matrix *L* in the system of equations:


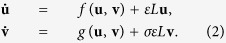


The matrix *L* represents the difference terms, with entries *L*_*i*,*j*_ = 1 if nodes *i* and *j* are connected, *L*_*i*,*j*_ = 0 otherwise, and *L*_*i*,*i*_ = −*k*_*i*_, where *k*_*i*_ is the *degree* of node *i*, such that each row sums to zero. The matrix *L* therefore describes diffusion in a system such that the flux to node *i* is given by the term: 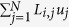.

## Diffusion on complex networks

In contrast to the continuous case, diffusion in discrete models can also take place along the edges of a more irregular underlying network. If the network has *N* nodes 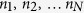, diffusion is mediated by the network Laplacian *L*, defined here in the same way as the regular case above. However, it must be noted that an alternative convention for network Laplacian is commonly used in areas of network science other than reaction-diffusion systems, whereby its sign is reversed, such that *L* = *D* − *A*, where *D* is the diagonal degree matrix and *A* is the adjacency matrix, defined in the same way as the off-diagonal elements above^16^.

Nakao and Mikhailov^5^ studied the Turing instability in large *Barabási–Albert*^9^ networks, which have a well known scale-free degree distribution on average. Their numerical investigations revealed the coexistence and multi-stability of a huge variety of patterns, as can be seen in Fig. 3 of their paper. They also found that stable patterns can exist before the homogeneous equilibrium becomes unstable, in a *subcritical* bifurcation. However, in related work it has been shown that the subcritical bifurcation from which the Turing instability originates can be made supercritical under the influence of feedback control^17^, or in fact from a change in parameters of the current system.

### The role of network structure and the optimal degree

Using an extension of a mean-field approach^18^, in order to understand the origin of patterns in this system, Wolfrum^15^ analysed a reduced system by considering the single-node dynamics. He studied the stability of an individual node of degree *k*_*i*_, with all other nodes considered fixed at the equilibrium 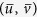, resulting in the single-node system:


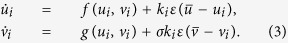


An analysis of the fixed-points of (3) reveals how the most basic single differentiated node (SDN) states bifurcate from the undifferentiated equilibrium-state. From this the lowest value of the parameter *σ* can be found where solutions exist, for any choice of *k*_*i*_. There is also a value of 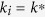 which itself results in the solution extending down to some minimum value of *σ*. This is referred to as the *optimal degree* and depends on the particular form of the functions *f* and *g* and choice of parameters therein. In this case, using the same system and parameters as Wolfrum (given in the Methods section) results in an optimal degree 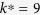. This reveals the interaction between the reaction kinetics and the node degree and shows an important connection between network structure and the dynamics of the system. In the current work we demonstrate that the relative position of the optimal degree nodes in the network hierarchy can affect the emergent behaviour of the system as a whole by modifying the solution structure.

#### Localised patterns and snaking solutions

As well as Turing patterns covering the whole spatial domain, spatially localised patterns have also been observed in a variety of experimental and numerical systems. In particular, localised buckling was observed in experiments on cylindrical shells by Hunt *et al*.^19^, and explained using numerical continuation using discretisation schemes. Numerical continuation and certain well-controlled experiments have shown that localised patterns of increasing spatial extent are connected by a winding structure in the solution structure known as snaking bifurcations. As the solution curves snake upwards in amplitude, the spatial pattern grows incrementally by one wavelength for each solution curve, as summarised by Hunt in his review of shell-buckling^20^. The origin and development of these localised solutions via snaking has now been extensively investigated in numerical and analytical studies of reaction–diffusion systems on regular lattices^21^. The mechanism for the transition between localised patterns via snaking is therefore theoretically well established for such regular systems.

Aside from reaction-diffusion systems, similar localised patterns have also been observed in physical experiments and bifurcation studies on other systems. Examples include experimental observations in optical systems^22^, numerical results for plain Couette flow^23^, as well as experimentally and analytically for hexagonal localised patterns in magnetic fluids^24^.

## Results

### From localised patterns to large-scale activation

In order to explore the growth of node-activity in networks and reveal the link to localised patterns in regular media, numerical continuation techniques were employed to track solutions in the parameter space (see Methods section). Starting from single differentiated node (SDN) solutions and following the solutions in the solution space transitions between the SDN states and those with multiply differentiated nodes (MDN) was investigated. The connection between different states in the system was uncovered, as well as their coexistence with the fully developed (“Turing”) patterns reported by Nakao and Mikhailov^5^.

### The growth of activation patterns

**Figure 1:**
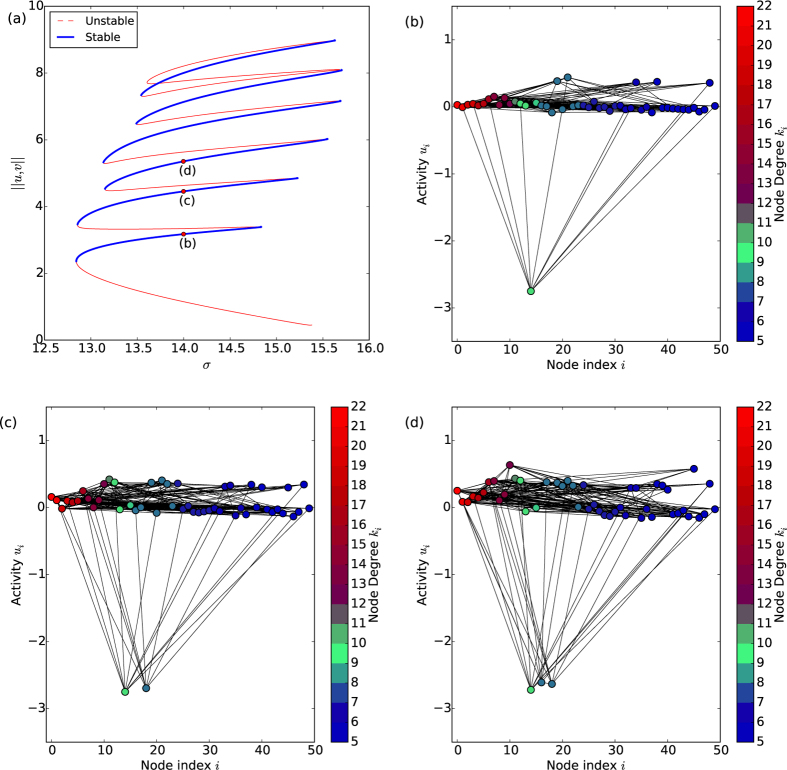
The growth of small-scale patterns of activity of nodes. (**a**) A bifurcation diagram showing the solution-space connecting patterns of increasing numbers of differentiated nodes. The control parameter is *σ* is plotted against the magnitude of the activation vector of the two species . Thick (blue) and thin (red) lines show stable and unstable solutions, respectively. The first three solutions are shown in (**b–d**), with nodes ordered left–right by decreasing node degree (*k*_*i*_ network neighbours – also shown in colour) and the lines show the links between adjacent nodes.

Figure 1 shows the result of following the solution from the ground-state (obtained using the numerical continuation techniques described in the Methods section), initially along an unstable branch which then becomes stable for a single excited node. It can be seen that, for certain cases, the solution curve exhibits a clear snaking behaviour, winding backwards and forwards under the influence of the parameter *σ*. Each of the bifurcation curves fold back to the left at some point, with the associated solutions becoming unstable as another node on the network becomes differentiated from the ground-state. This is directly analogous to the snaking behaviour seen in regular topologies as larger patterns develop from more localised ones. In Fig. 1(a) each stable branch of the solution curve corresponds to a different activation state with different numbers of differentiated nodes (three of which are shown in the node activation diagrams Fig. 1(b–d)), corresponding to larger values of the magnitude (L-2 Norm) of the vector displacements 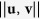, plotted on the *y*-axis. In the example shown, with a clear snaking structure, the branches connect solutions with increasing numbers of differentiated nodes, the rest of the network remaining largely the same (see supplementary video snaking.mov). This universal snaking behaviour therefore appears to provide a strong connection to localised pattern formation and opens up the possibility of applying this well developed theory to problems in random networks.

The solutions can be continued further, with the solution structure becoming highly complicated. However, for this network realisation, where the optimal degree (shown in green) is somewhere part-way between the core and periphery of the network, the bifurcations do not directly connect the patterns of “localised” activity to the bulk “Turing-type” modes. These modes are abundant, but exist in a disconnected subset of the turmoil of solutions.

#### Development of large scale activation

**Figure 2:**
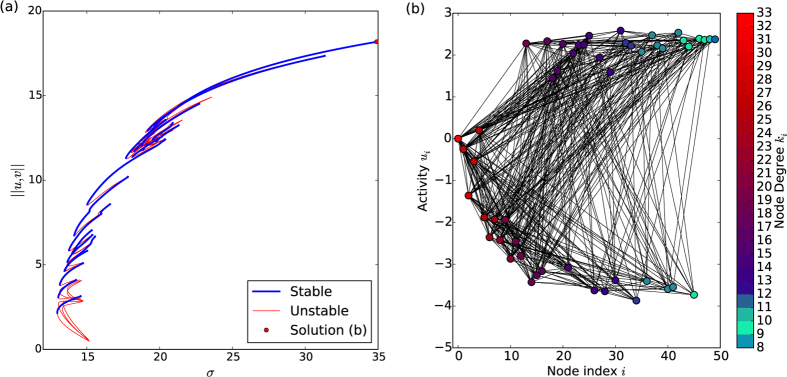
Growth from localised activity to system scale *Turing-type* patterns in a network with *attachment degree M* = 10. (**a**) The snaking bifurcation diagram, with solutions at turning points numbered and (**b**) a bulk-mode pattern on the network nodes. The optimal degree nodes (shown in green) are towards the periphery of the network.

Different network structures can effect different system behaviour, whereby the localised states do indeed connect to the Turing modes of full-blown system-scale activation. For this alternative network realisation a degree of attachment of *M* = 10 was used to generate the network. In this case the optimal degree 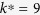 nodes were instead found towards the periphery of the network, being amongst the lowest degree. The connection between the two regimes is again via a complicated snaking structure, as shown in the example in Fig. 2, where the “snakes” are found to wind up and down as different nodes become differentiated and undifferentiated. This provides a clear connection between the small-scale patterns analysed by Wolfrum and the larger bulk patterns of activity seen in the work of Nakao and Mikhailov. The difference between this and the previous case reveals the importance of the network structure, its interaction with the reaction dynamics and the resulting behaviour of the system. The position of the optimal degree nodes within the network strongly affects the connectedness of solutions as these are preferentially the first nodes to be activated.

#### Multiplicity of solutions

It is clear that at there exist an enormous number of coexisting combinations of differentiated nodes, connected by a vast number of complicated structures in the solution space, covering a range of parameter values. Understanding the statistical distribution of states over these values will be valuable in understanding the variety of patterns and possible configurations that can be exhibited by such systems.

**Figure 3:**
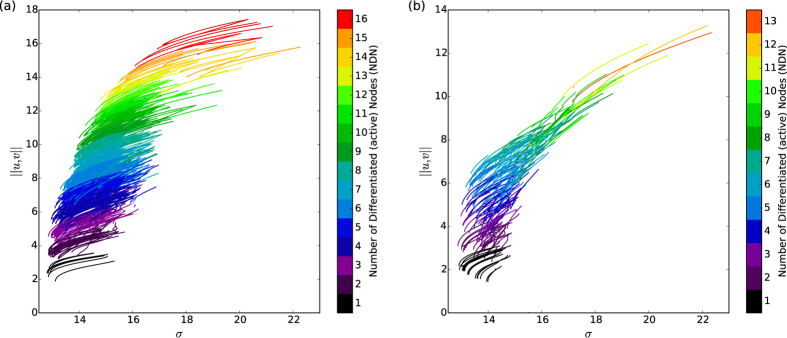
Full set of solution branches at lower *σ* values, showing both stable and unstable branches together. Colours indicate the number of differentiated nodes on each curve. (**a**) The *M* = 5 networks with intermediate in position, and (**b**) the *M* = 10 network with near the periphery.

The numerical continuation methods used in this work can be used to uncover larger portions of this structure and give insight into the level of complexity likely to exist under different conditions, forced by the external driving parameters. As before, the initial SDN solutions were isolated before being continued in the parameter space, as can be seen in the lower branches of Fig. 3. The region that these solutions occupy closely agrees with the predictions of the reduced-system approach of Wolfrum. In addition, the stable branches that appear for for the lowest value of *σ* are those with the optimum degree, as predicted from Wolfrum’s work. Each of these SDN states were then used as initial conditions for the continuation further up into MDN solutions. The solutions for all accessible states with up to 9DN are shown together in Fig. 3. These different solutions coexist multistably at the same parameter values. The clear bunching seen for SDN solutions becomes more diffuse as more nodes become differentiated and the nodes interact more strongly. Solutions are tightly bounded in the bifurcation space and contained in a region which continues to high values of *σ* and bulk modes of activity.

A notable feature of the results is that the column of solutions curves markedly to the right. This can be explained heuristically by considering that, for each number of differentiated nodes there exists some stable branch that appears for minimal *σ*, which consists of solutions with differentiated nodes of optimal degree, as predicted by Wolfrum^15^. However, at some point he MDNs run out of nodes with optimal degree to be differentiated and instead another node activates, which necessarily has a higher activation *σ*. Determining these *regions of existence* for networks should be amenable to analytical treatment and provide an important area for future investigation in such complex systems.

#### Statistical density of states

**Figure 4:**
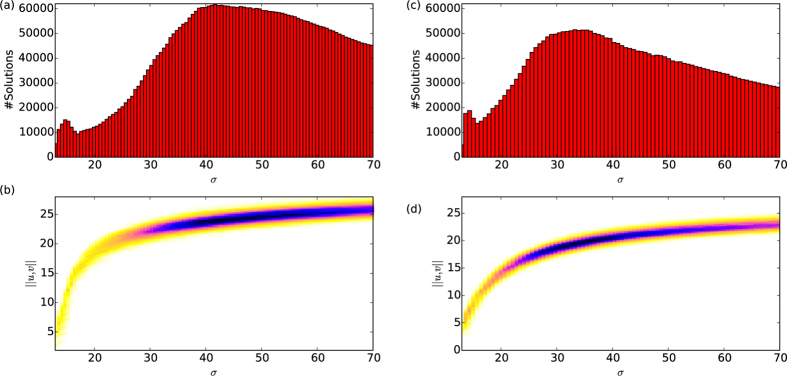
Statistical distributions of solutions, shown as the both histogram and 2D density plot over the bifurcation space for a range of values of the control parameter *σ* (**a**,**b**) are for the *M* = 5 case where the small-scale patterns do not directly connect to the system-scale patterns and (**c**,**d**) for the *M* = 10 case where they do connect. In both cases two peaks can be seen, one for the snaking column and the other for the bulk solutions.

Thousands of realisations were initiated and continued in order to produce a statistical description of the state density at different parameter values. Two clear peaks can be seen in the histograms of Fig. 4(a,c), with the broader of the two peaks appearing at higher *σ* associated with bulk modes of the system. The narrow peak at low *σ* is associated with the cluster of localised solutions and the growth to extended patterns, connected via the snaking bifurcations. Clear differences can be seen for the two different network topologies, again highlighting the important role of network structure in organising the dynamics. In the more highly connected *M* = 10 network the bulk solutions are most abundant at lower values of the control parameter *σ* and are more smoothly connected than in the case where the optimal degree is buried deep in the network. The region of existence described in the previous section can be most clearly seen in the two dimensional density plot shown in Fig. 4(b,d), showing the density of bifurcation curves over a wide range of the parameter *σ*. The projection onto a one-dimensional histogram in *σ* shown in (b) clearly shows the number of available system configurations at each of the parameter values.

These results reveal the potential states accessible to this system, and similar treatment could shed light on the potential configurations possible in a vast number of systems of this type arising in nature. Examples of such systems with both activator-inhibitor kinetics as well as complex interaction topologies include: gene networks; protein species interaction networks, such as those seen in early evolution; and competition networks in other complex natural and human systems, such as social and economic systems.

## Discussion

In this research a reaction diffusion system on a complex network topology has been numerically investigated in detail for symmetric networks with a particular reaction dynamics, with generally applicable methods and results. The results have revealed the transition between the single-node solutions analysed by Wolfrum, which originate from the undifferentiated state, and the fully developed patterns reported by Nakao and Mikhailov. This was carried out by numerical continuation of the solutions in the parameter-space of the system and study of the solutions found along the various multistable branches found at each set of values. The states of the system have a snaking solution structure, showing a deep theoretical connection to localised patterns seen in reaction-diffusion systems on regular topologies. The universality of this snaking behaviour therefore opens up the possibility of applying the well developed theory of homoclinic snaking to problems in random networks. Through this the results reveal the origin of and connection between the multistability of states found previously.

A statistical analysis of the solution-structure has been used to present the multiplicity of configurations available to systems on networks. Understanding the density of solutions and bifurcation curves that exist within certain regions of the control parameter space is expected to reveal much about the underlying systems being modelled. In systems of competing chemical or biological agents the density of states could indicate the diversity of species that can exist at different values of some external environmental parameter. Similar analogies could be made when modelling social systems, as well as many other complex systems, as competing interactions via networks.

The important connection between network structure, reaction kinetics and resulting system behaviour has also been demonstrated, including the importance of the optimal degree nodes discovered by Wolfrum. This adds to the growing interest in the interplay between network structure and dynamics that have been investigated in the context of ecological networks and elsewhere^25^,^26^,^27^. Already in-roads have been made into other theoretical aspects of related systems such as multiplex networks^28^, including generalisations of Wolfrum’s original analysis to such systems^29^. Other research has investigated oscillatory Turing patterns (originating from a so called “wave bifurcation”) for symmetric networks in the context of ecological networks^30^, as well as directed networks^31^. The current work provides insight into the nature of emergent patterns of activity at different scales in networks of interacting species and, by connecting various areas of research, hopes to open the field up to deeper study.

## Methods

### The Mimura-Murray model

The current investigation focusses on the Mimura-Murray model of prey-predator populations^32^, following on from the work of Nakao and Mikhailov^5^. The kinetics of the reaction-diffusion system (eq. (2)) are described by the following equations:


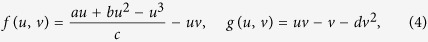


where *u* denotes the activator (or prey) and *v* the inhibitor (or predator). In these investigations *σ* is the bifurcation (control) parameter, related to the relative strengths of the diffusion terms. The current study was carried out using the values *a* = 35, *b* = 16, *c* = 9, *d* = 2/5, *ε* = 0.12, at which the system possess the equilibrium 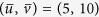. This *undifferentiated* state is stable for small values of *σ* but loses stability in a *subcritical* bifurcation at *σ* ≈ 15.5. In continuous media this would result in the emergence of alternating activator-rich and activator-low domains (a periodic *Turing-type* pattern in the supercritical case), but organised on networks can also display small-scale (“localised”) patterns of node activity in these subcritical cases. Using the current parameter values in the reaction functions (4), linear stability analysis of equation (3) results in an optimal degree of 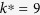, which is shown in this work to play a key role in organising the behaviour of the system.

### Network properties

In order to investigate the development and growth of “patterns” of activity on non-regular network topologies, the system was set up with the reaction species (on the nodes) interacting via the edges of a *Barabási–Albert*^9^ network.

**Figure 5:**
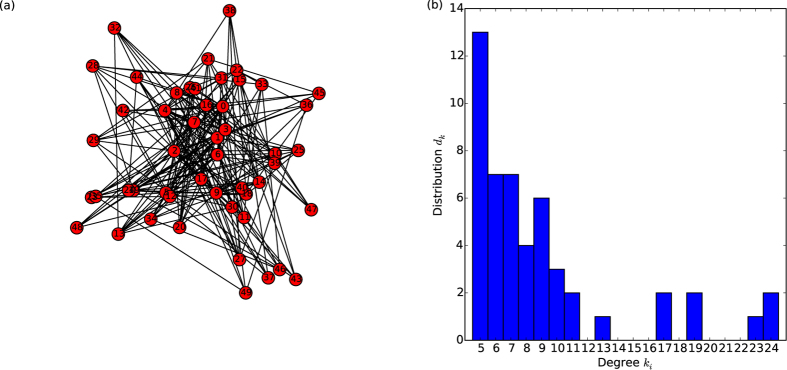
An example network used in this investigation (**a**), using the preferential attachment scheme of Barabási and Albert (BA). The number of nodes is *N* = 50 and degree of attachment for each newly added node is *M* = 5. (**b**) The degree distribution for the network used in the first network reported in this work. The BA scheme is known to produce scale-free degree distributions on average and in the limit of *N* → ∞ but here, where *N* is finite, it is only approximately scale-free.

A network with 50 nodes was used for these investigations, where new nodes added at each generation step have five edges assigned preferentially to higher degree existing nodes. The routines in the *NetworkX* module for Python were used to generate and visualise the networks. Different topologies with the same characteristics were investigated to ensure consistent qualitative behaviour, but only one representative realisation is shown here. For the majority of results in this work the *degree of attachment M* = 5 was used in the network generation scheme (Fig. 5). In these cases it can be seen that numerous nodes of the optimal 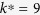 lie towards the middle of the degree distribution. For the later (comparison) cases *M* = 10 was used to generate the network, in which the optimal degree nodes instead have amongst the lowest degree in the network (being close to the *periphery*), resulting in important differences in the system behaviour.

### Numerical techniques

Computations were started from the single-node (SDN) solutions, studied in previous work^15^, which bifurcate from the undifferentiated state at the point *σ*_*T*_. All possible singly differentiates node (SDN) solutions were found by numerically integrating the equations of the system from some random initial condition then refining using numerical root finding (using the widely available fsolve routine). The solutions were then followed back and forth in their meander through the parameter space of the system using numerical *continuation* techniques provided by the AUTO bifurcation software^33^.

## Additional Information

**How to cite this article**: McCullen, N. and Wagenknecht, T. Pattern Formation on Networks: from Localised Activity to Turing Patterns. *Sci. Rep.* **6**, 27397; doi: 10.1038/srep27397 (2016).

## Supplementary Material

Supplementary Information

Supplementary Information
