# Supplementary material for: Pattern Formation on Networks: from Localised Activity to Turing Patterns
Source: Sci Rep. 2016 Jun 7;6:27397. doi: 10.1038/srep27397 (PMC4895380; doi:10.1038/srep27397)
Supplement: Supplementary Information [file srep27397-s2.pdf]

Pattern Formation on Networks: from Localised Activity to Turing Patterns

Nick McCullen[1,*] **and Thomas Wagenknecht**[2, 3]

[1]Centre for Networks and Collective Behaviour, Architecture & Civil Engineering, University of Bath, UK.
[2]Department of Applied Mathematics, University of Leeds, UK.
[3]Deceased
[*]n.mccullen@physics.org

Supplementary video

Parametric growth of activation patterns.

The supplementary video shows the continuation of the solutions using the numerical techniques described in Methods. The top panel shows the magnitude (the L2-Norm $||\mathbf{u},\mathbf{v}||$) of the coexisting solutions over a range of the parameter σ. The bottom panel shows the activation of the nodes on the network (ordered by node degree) along the solution branches as the pattern develops. The first excitation of a single node is initially an unstable solution (dashed lines in the solution bifurcation diagram), becoming stable (solid lines) at a turning point, before winding backwards and forwards under the influence of σ. As each bifurcation curve folds back to the left the associated solution becomes unstable and another node on the network becomes activated.
